# Effect of crystal-to-detector distance shift on data processing in serial crystallography

**DOI:** 10.1371/journal.pone.0327019

**Published:** 2025-06-26

**Authors:** Ki Hyun Nam

**Affiliations:** College of General Education, Kookmin University, Seoul, Republic of Korea; Chung-Ang University, KOREA, REPUBLIC OF

## Abstract

Serial crystallography (SX) is an emerging technique to determine the room-temperature structure of macromolecules while minimizing any radiation damage. In SX, many crystals are continuously delivered to the X-ray interaction points. Crystal-to-detector distance (CTDD), an important data processing parameter, changes during sample delivery. However, the ways in which these variations affect the data quality remain unclear. Therefore, this study aimed to assess the impact of CTDD accuracy on SX data quality. Serial synchrotron crystallographic data of hen egg white lysozyme (HEWL) and glucose isomerase (GI) were processed at various CTDDs using three indexing algorithms. The CTDD that yielded the maximum number of indexed images differed from the optimized CTDD, and the resulting indexing trends varied depending on the algorithm used. The indexed data near the actual CTDD exhibited a Gaussian unit cell distribution pattern; however, this pattern became distorted as the CTDD deviated from the actual CTDD value, resulting in changes in the indexed unit cell dimensions. The phase problem was successfully solved even when HEWL and GI data deviated by ±10 mm from the actual CTDD, excluding one dataset. For HEWL processed via MOSFLM, structure refinement statistics maintained acceptable R_free_ values despite a ± 10 mm deviation; however, data quality deteriorated with increasing deviation from the actual CTDD. Collectively, these results provide valuable insights for efficient SX data processing and interpretation.

## Introduction

Serial crystallography (SX), which uses X-ray free-electron lasers or synchrotron X-rays, facilitates the determination of the room-temperature structures of macromolecules while minimizing any radiation damage [1–6]. It also provides biologically relevant structural information, including conformation and molecular flexibility data [6–9]. SX with a pump-probe or mixing-and-injection system facilitates the visualization of time-resolved molecular dynamics, providing insights into key molecular functions [10–16].

Generally, a crystal is exposed to X-rays only once in SX [17,18]. Therefore, diffraction data of numerous crystals must be collected to obtain the complete three-dimensional structural information [19–21]. During SX data collection, many crystals are delivered to the X-ray interaction point using various sample delivery systems, such as the injection, fixed-target scanning, and hybrid systems [22–30]. During sample delivery, crystal position can change with respect to the X-ray beam. For example, in an injector system, if the width of the injection stream is several hundred micrometers, the crystal sample can be positioned randomly within that range [31], indicating variations in the crystal-to-detector distance (CTDD). Additionally, low stability of the injection stream and sample shifts along the direction of the X-ray beam change the CTDD. In fixed-target scanning, crystal position can be changed according to the thickness of the sample holder. If the sample holder is not perfectly aligned vertically to the X-ray beam, CTDD will vary during the horizontal or vertical scanning of the sample holder. Therefore, crystal position at the X-ray interaction point may not be perfectly identical in experiments where several crystals are delivered during SX data collection. In addition to the detector movement, which is often non-reproducible, injection system position is also frequently non-reproducible upon reinsertion.

During SX data processing, prior information on indexing parameters, such as X-ray energy, detector information, and CTDD, is essential to process the Bragg peaks [32,33]. Additionally, accurate information on the experimental geometry is crucial for high indexing success and generation of reliable structural information [32,34,35]. Among various geometric parameters, accurate CTDDs are essential for the precise indexing and integration of Bragg peaks. Typically, small-molecule powders (e.g., LaB_6_ and CeO_2_) [18] and protein crystal samples are used in SX experiments as references to obtain accurate CTDDs [36]. Although these methods provide reliable physical information on CTDDs, inaccurate CTDD measurement can adversely affect the data quality and structural accuracy.

In this study, CTDD variation tolerance was investigated using the serial synchrotron crystallography (SSX) diffraction data of hen egg white lysozyme (HEWL) and glucose isomerase (GI), which is widely used as a standard crystal in macromolecular crystallography and SX, with data processed using the CrystFEL program. Notably, number of indexed patterns and indexable range of CTDD varied depending on the indexing algorithm. Furthermore, this study extensively analyzed the statistics of data processing, molecular phasing, and structure determination using various input CTDDs. The experimental results provided insights into CTDD-related data processing in SX.

## Materials and methods

### Sample preparation

HEWL was purchased from Hampton Research (Cat. No. HR7–110; Aliso Viejo, CA, USA). It was crystallized as previously described [37]. Briefly, 50 mg/mL HEWL and crystallization (0.1 M sodium acetate [pH 4.0], 6% [w/v] polyethylene glycol 8000, and 2 M NaCl) solutions were mixed in a microcentrifuge tube. After vortexing, the mixture was stored at 20 °C. HEWL crystals were formed within 30 min with a dimension of 20–30 μm.

### Data collection

SX diffraction data were collected at the 11C beamline of Pohang Light Source II (Republic of Korea) [38]. X-ray wavelength was 0.9794 Å (12.659 keV), with a photon flux of approximately 1 × 10^12^ photons/s. The sample-to-detector distance was set to 300 mm. During SSX data collection, the center of the injection stream was aligned in the vertical direction of the goniometer head. HEWL crystals were delivered to the X-ray interaction point using the syringe and syringe pump method, as previously described [39]. HEWL was embedded in a polyacrylamide (PAM) injection matrix [40] as a viscous medium in a dual-syringe setup [17]. Then, an injector containing the crystals embedded in the PAM injection matrix was installed on the Fusion Touch 100 syringe pump (CHEMYX, Stafford, TX, USA). Crystal samples were extruded from the syringe using a syringe pump-based sample delivery system at a flow rate of 100 nL/min [31]. The center of the injection stream was aligned to the X-ray beam path using an inline camera installed in the experimental hutch. Diffraction data were collected at 25 ± 0.4°C and recorded on the Pilatus 6M detector (DECTRIS, Baden, Switzerland).

### Data processing

For HEWL, hit images containing Bragg peaks were filtered using Cheetah [41]. X-ray diffraction images were processed using CrystFEL [42] with the MOSFLM [43], DirAx [44], and XGANDALF [45] indexing algorithms. An indexing method was used with the “nolatt-nocell” parameter. Detector geometry was optimized using *geoptimiser* [35] in CrystFEL. For GI, previously reported diffraction images were obtained from the Coherent X-ray Imaging Data Bank [46], and data was processed with the same method used for HEWL. During data indexing, unit cell size (a = b = 79.30 Å, c = 37.73 Å, and α = β = γ = 90° for HEWL; a = 94.54 Å, b = 100.36 Å, c = 103.59 Å, and α = β = γ = 90° for GI) was enforced. Tolerance for unit cell information was set to the default values, with a = b = c = 5% and α = β = γ = 1.5%. During the indexing of Bragg peaks, CTDD input in the geometry file was 288–308 mm for HEWL and 238–258 mm for GI. During indexing, CTDD input was adjusted in 1-mm increments in all distance ranges. Unit cell distribution was visualized using *cell_explore* implemented in CrystFEL.

### Structure determination

Phase problem was solved via molecular replacement (MR) using the Phaser-MR program implemented in PHENIX [47]. Crystal structures of HEWL (Protein Data Bank code: 6IRJ) [48] and GI (PDB code: 7CVK) [46] were used as search models. Structural refinement was performed using phenix.refine in PHENIX [47], with default parameters. Water molecules were automatically added to the model structure during structural refinement. The final structure was validated using MolProbity [49], and structural figures were generated using PyMOL (http://pymol.org).

## Results

### Data collection and indexing

For HEWL data collection via SSX, inner diameter of the syringe needle was 163 μm, and width of the PAM injection stream, including the HEWL crystals, was approximately 500 μm. Increase in the injection stream width from the inner diameter of the syringe needle is due to the low viscosity of the PAM injection matrix at a low flow rate [31]. In the experimental setup, injection stream was designed to pass through a location where the CTDD was 300 mm; however, the actual optimized distance processed by CrystFEL was approximately 298.5 mm. These distance differences were due to experimental errors that occurred when the injection stream was manually aligned horizontally to the X-ray interaction region. Considering the distribution of the HEWL crystals within the PAM injection matrix, theoretical CTDD was estimated to be approximately 298.5 ± 0.25 mm.

**Fig 1:**
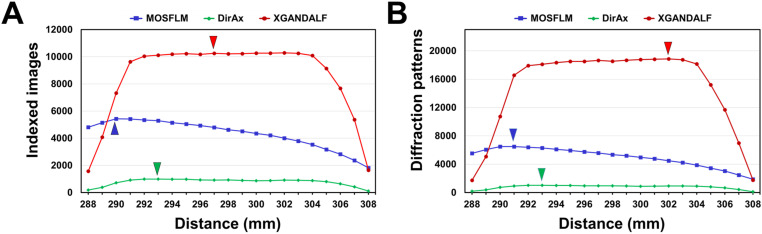
Indexing of the serial synchrotron crystallography (SSX) diffraction images of the hen egg white lysozyme (HEWL) processed using the MOSFLM, DirAx, and XGANDALF algorithms at various crystal-to-detector distance (CTDDs). **(A)** Number of indexed images of HEWL. **(B)** Number of diffraction patterns including multicrystal hits from the indexed images of HEWL. Maximum numbers of indexed images and multicrystal diffraction patterns processed by MOSFLM (blue), DirAx (green), and XGANDALF (red) are indicated by triangles.

During data collection, 12,082 hit images containing Bragg peaks were filtered and processed. Indexing efficiency of diffraction images varies depending on the indexing algorithm [50]. Therefore, HEWL data were processed using the MOSFLM, DirAx, and XGANDALF algorithms to compare how sensitive each algorithm is to CTDD deviations during diffraction image processing. For HEWL data processed using MOSFLM, maximum number of indexed diffraction images was 5,428 at a CTDD of 290 mm, with an indexing rate of 44.92% (Fig 1A). The number of indexed images decreased below and above this CTDD. Compared to the maximum number of images at 290 mm, indexing rate remained over 90% at 289–296 mm (Fig 1A). During SX data collection, multiple crystals can be simultaneously exposed to X-rays during sample delivery, resulting in multicrystal diffraction patterns being recorded in a single image. The multicrystal hit rate, defined as the number of indexed diffraction patterns divided by the total number of images, exhibited a similar trend, reaching a maximum number of images of 6481 at a CTDD of 291 mm, with a multicrystal hit rate of 119.39%, and gradually decreasing below and above this CTDD (Fig 1B). A multicrystal hit rate above 100% indicates that multiple crystal diffraction patterns were indexed from a single diffraction image, a situation that often occurs when the crystal concentration is high.

Maximum number of indexed diffraction images for the HEWL data processed using DirAx was 994 at a CTDD of 293 mm, resulting in an indexing rate of 8.22% (Fig 1A). At CTDDs of 290, 289, and 288 mm, numbers of indexed images were 724, 388, and 186, respectively, showing a marked decrease from the maximum indexing rate as the CTDD decreased. Conversely, in the CTDD range of 294–304 mm, number of indexed images exceeded 869, showing a gradual decrease from the maximum indexing rate as the CTDD increased. The multicrystal hit rate followed a similar trend, reaching a maximum of 1020 images at a CTDD of 291 mm, with a multicrystal hit rate of 102.61% at 293 mm, and gradually decreasing below and above this CTDD (Fig 1B).

For HEWL data processed using XGANDALF, maximum number of indexed diffraction images was 10,249 at a CTDD of 297 mm, resulting in an indexing rate of 84.82% (Fig 1A). Indexing rate remained above 80% at CTDDs of 291–304 mm. However, it fell below 50% at < 289 and > 307 mm CTDDs and below 14% at 288 and 308 mm CTDDs. The multicrystal hit rate also showed a similar trend, reaching a maximum number of images of 18873 at a CTDD of 302 mm, with a multicrystal hit rate of 183.46%. The multicrystal hit rate was above 170% in the CTDD range of 291–304 mm (Fig 1B).

Notably, data processing results at different CTDDs can change depending on the crystal form, diffraction quality, and experimental setup. To better understand the effect of CTDD on data quality, additional data processing was performed using a previously reported GI dataset collected via fixed-target SSX [46]. In total, 15,308 GI diffraction images were used in this study, and the previously optimized CTDD was approximately 248.82 mm.

**Fig 2:**
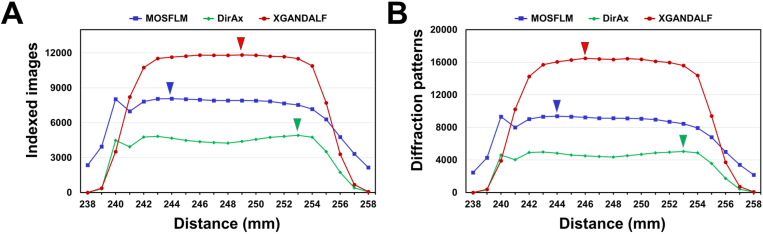
Indexing of the serial synchrotron crystallography (SSX) diffraction images of the glucose isomerase (GI) processed using the MOSFLM, DirAx, and XGANDALF algorithms at various crystal-to-detector distance (CTDDs). **(A)** Number of indexed images of GI. **(B)** Number of diffraction patterns including multicrystal hits from the indexed images of GI. Maximum numbers of indexed images and multicrystal diffraction patterns processed by MOSFLM (blue), DirAx (green), and XGANDALF (red) are indicated by triangles.

For GI data processed using MOSFLM, maximum number of indexed diffraction images was 8,072 at a CTDD of 244 mm, with an indexing rate of 52.73% (Fig 2A). Number of indexed images decreased below and above this CTDD. Compared to the maximum number of images at 244 mm, indexing rate remained over 85% at 240–254 mm but decreased to less than 50% at < 239 mm or > 257 mm CTDDs (Fig 2A). The multicrystal hit rate followed a similar trend, reaching a maximum number of images of 9380 at a CTDD of 244 mm, with a multicrystal hit rate of 116.20%, and gradually decreasing below and above this CTDD (Fig 2B).

For GI data processed using DirAx, maximum number of indexed diffraction images was 4,915 at a CTDD of 253 mm, resulting in an indexing rate of 32.10% (Fig 2A). At CTDDs of 238, 239, 257, and 258 mm, numbers of indexed images were 2, 326, 412, and 52, respectively, indicating a marked decrease from the maximum indexing rate as the CTDD decreased. Conversely, in the CTDD range of 240–254 mm, number of indexed images exceeded 3,938, indicating a gradual decrease from the maximum indexing rate as the CTDD increased. The multicrystal hit rate showed a similar trend, reaching a maximum of 5047 images at a CTDD of 253 mm, with a multicrystal hit rate of 102.68%, and gradually decreasing below and above this CTDD (Fig 2B).

For GI data processed using XGANDALF, maximum number of indexed diffraction images was 11,838 at a CTDD of 249 mm, resulting in an indexing rate of 77.33% (Fig 2A). At CTDDs of 242–354 mm, indexing rate remained above 70%. However, the indexing rate fell below 50% at < 240 and > 256 mm CTDDs and below 5% at < 239 and > 257 mm CTDDs. Additionally, multicrystal hit rate followed a similar trend, reaching a maximum number of images of 16481 at a CTDD of 246 mm, with a multicrystal hit rate of 138.42% (Fig 2B).

Taken together, number of indexable diffraction patterns and trends in indexing range at specific CTDDs vary depending on the indexing algorithm. The CTDDs that yielded the maximum number of indexed images of HEWL and GI processed using XGANDAFL were similar to the optimized CTDDs processed using CrystFEL, whereas those that yielded the maximum number of indexed images of HEWL and GI processed using MOSFLM and DirAx differed significantly from the optimized CTDDs.

### Unit cell distribution

SX data were indexed using the known lattice parameters of HEWL (a = b = 79.30 Å, c = 37.73 Å, and α = β = γ = 90°). The crystal lattice tolerance during processing was set to the default values (a = b = c = 5% and α = β = γ = 1.5%) in the CrystFEL program. Consequently, the collected lattice parameters covered a broader range than the input values of the unit cell dimensions. Theoretically, unit cell dimensions for indexing allow for a = b = 79.30 ± 3.9650 Å, c = 37.73 ± 1.8865 Å, and α = β = γ = 90 ± 1.35°. Distribution of the unit cell parameters for the indexed diffraction patterns was investigated to analyze the correlation between the maximum-indexed CTDD and unit cell distribution.

**Fig 3:**
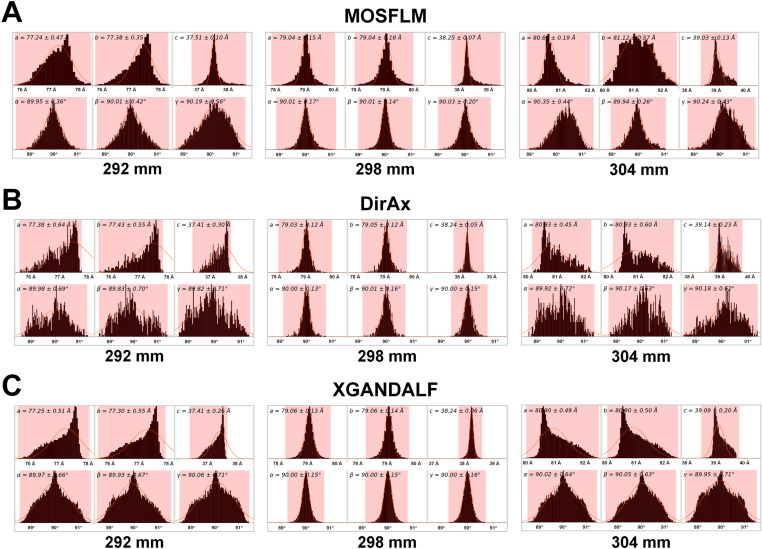
Unit cell distribution of the indexed HEWL images processed by **(A)** **MOSFLM,** **(B)** **DirAx, and** **(C)** **XGANDALF at various input CTDDs.** The histogram shows the distribution of unit cell parameters (a, b, c, α, β, and γ) extracted from indexed diffraction patterns. At a CTDD of 298 mm, which is close to the actual CTDD value, the unit cell parameters exhibit a clear Gaussian distribution. In contrast, CTDD values of 292 mm and 304 mm, which deviate from the actual value, result in distorted Gaussian distributions of the unit cell parameters.

For HEWL data processed using MOSFLM, unit cell distribution for CTDDs of 297–299 mm showed a typical Gaussian pattern (Fig 3A; S1 Fig). At a CTDD of 298 mm, deviation in the unit cell parameters was minimal, with unit cell dimensions of a = 79.04 ± 0.15 Å, b = 79.04 ± 0.18 Å, c = 38.25 ± 0.07 Å, α = 90.01 ± 0.17°, β = 90.01 ± 0.14°, and γ = 90.03 ± 0.20°. At 292 mm, where the near-maximum number of indexed images was generated using MOSFLM, unit cell distribution exhibited a distorted Gaussian pattern (Fig 3A).

For HEWL data processed using DirAx, a typical Gaussian unit cell distribution was observed at CTDDs of 298–299 mm (Fig 3B; S2 Fig). At a CTDD of 298 mm, deviation in the unit cell parameters was minimal, with unit cell dimensions of a = 79.03 ± 0.12 Å, b = 79.05 ± 0.12 Å, c = 38.24 ± 0.05 Å, α = 90.00 ± 0.13°, β = 90.01 ± 0.16°, and γ = 90.00 ± 0.15°. At 292 and 294 mm, corresponding to the near-maximum image-indexed CTDDs, unit cell distribution did not exhibit a Gaussian pattern (Fig 3B; S2 Fig).

For HEWL data processed using XGANDALF, unit cell distributions at CTDDs of 298 and 299 mm exhibited a typical Gaussian pattern (Fig 3C; S3 Fig). At a CTDD of 298 mm, deviation in the unit cell parameters was minimal, with unit cell dimensions of a = 79.06 ± 0.13 Å, b = 79.06 ± 0.14 Å, c = 38.24 ± 0.06 Å, α = 90.00 ± 0.15°, β = 90.00 ± 0.15°, and γ = 90.00 ± 0.16°. At 302 mm, corresponding to the maximum image-indexed CTDD, unit cell distribution did not exhibit a Gaussian pattern (S3 Fig).

SX data of GI were indexed using the known lattice parameters of GI (a = 94.54 Å, b = 100.36 Å, c = 103.59 Å, and α = β = γ = 90°). Crystal lattice tolerance during processing was set to the default values in the CrystFEL program. Theoretically, unit cell dimensions for indexing were a = 94.54 ± 4.727 Å, b = 100.36 ± 5.018 Å, c = 103.59 ± 5.1795 Å, and α = β = γ = 90 ± 1.35°. Subsequently, unit cell parameter distribution in the indexed diffraction patterns was investigated at various input CTDDs.

**Fig 4:**
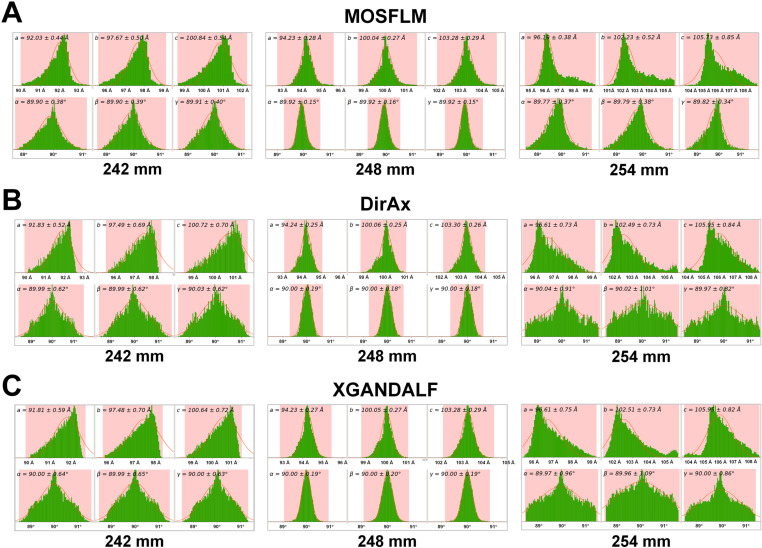
Unit cell distribution of the indexed GI images processed by **(A)** **MOSFLM,** **(B)** **DirAx, and** **(C)** **XGANDALF at various input CTDDs.** The histogram shows the distribution of unit cell parameters extracted from indexed diffraction patterns. At a CTDD of 248 mm, which is close to the actual CTDD value, the unit cell parameters exhibit a clear Gaussian distribution. In contrast, CTDD values of 242 mm and 254 mm, which deviate from the actual value, result in distorted Gaussian distributions of the unit cell parameters.

For GI data processed using MOSFLM, unit cell distributions at CTDDs of 248 and 249 mm displayed a typical Gaussian pattern (Fig 4A; S4 Fig). At a CTDD of 248 mm, deviation in the unit cell parameters was minimal, with unit cell dimensions of a = 94.23 ± 0.28 Å, b = 100.04 ± 0.27 Å, c = 103.28 ± 0.29 Å, α = 89.92 ± 0.15°, β = 89.92 ± 0.16°, and γ = 89.92 ± 0.15°. At 244 mm, where the maximum number of indexed images was generated using MOSFLM, unit cell distribution exhibited a distorted Gaussian pattern (S4 Fig).

For GI data processed using DirAx, unit cell distributions at CTDDs of 248 and 249 mm showed a typical Gaussian pattern (Fig 4B; S5 Fig). At a CTDD of 247 mm, deviation in the unit cell parameters was minimal, with unit cell dimensions of a = 93.86 ± 0.21 Å, b = 99.65 ± 0.20 Å, c = 102.88 ± 0.23 Å, α = 89.99 ± 0.14°, β = 90.00 ± 0.15°, and γ = 90.00 ± 0.14°. At 252 and 254 mm, corresponding to the near-maximum image-indexed CTDD, unit cell distribution exhibited a distorted Gaussian pattern (S5 Fig).

For GI data processed using XGANDALF, the unit cell distribution for the CTDDs of 248–249 mm displayed a typical Gaussian distribution (Fig 4C; S6 Fig). At a CTDD of 249 mm, the deviation in the unit cell parameters was minimal, with unit cell dimensions of a = 93.85 ± 0.23 Å, b = 99.64 ± 0.23 Å, c = 102.86 ± 0.26 Å, α = 90.00 ± 0.16°, β = 90.00 ± 0.16°, and γ = 90.00 ± 0.16°. At 249 mm, which corresponded to the maximum image-indexed CTDD, the unit cell distribution followed a Gaussian pattern (S6 Fig).

Taken together, unit cell distributions of HEWL and GI data processed using the MOSFLM, DirAx, and XGANDALF algorithms commonly exhibited Gaussian pattern within the CTDD ranges of 298–299 and 248–249 mm, respectively, which were close to the actual optimized CTDD processed using CrystFEL. When the CTDD was greater than the optimized CTDD, unit cell distribution exhibited a distorted Gaussian pattern. Notably, unit cell dimensions a, b, and c changed as the CTDD deviated from the optimized CTDD value. Additionally, unit cell dimensions decreased or increased when the CTDD was less or greater than the optimized value, respectively.

### Statistics of data processing

To investigate the effect of CTDD on data quality, all indexed data were processed. For HEWL data, CC1/2 dropped at resolutions below 1.8 Å at the optimal CTDD. Therefore, the dataset was processed up to 1.8 Å. For GI data, resolution cutoff was set at 1.7 Å based on previous data processing results.

**Fig 5:**
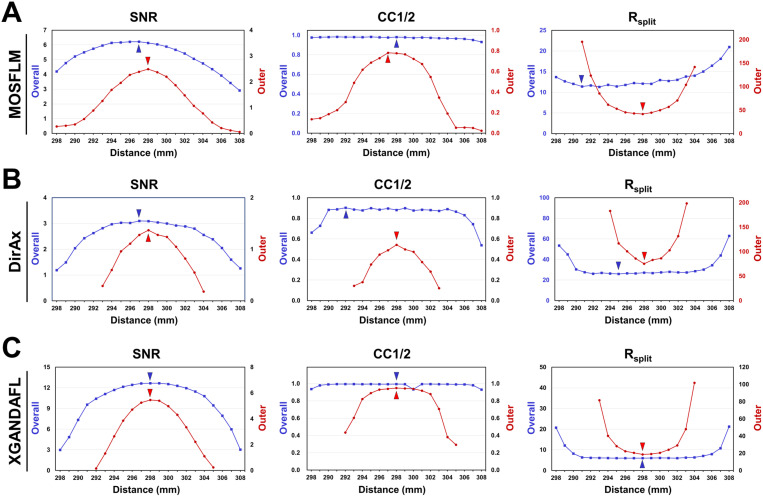
Statistics of HEWL data processing using different algorithms at various input CTTDs. **(A)** MOSFLM. **(B)** DirAx. **(C)** XGANDALF. Overall (■; blue lines) and outer shell (●; red lines) resolution ranges were 80–1.80 and 1.86–1.80 Å, respectively. Data points with an SNR less than 0 or an Rsplit greater than 200 were excluded from the data plot.

For HEWL data processed using MOSFLM, highest overall signal-to-noise ratio (SNR) and R_split_ values were 6.22 and 12.26%, respectively (Fig 5A), both obtained from the data processed at a CTDD of 297 mm, which differed slightly from the actual CTDD. The highest overall half correlation coefficient (CC1/2) was 0.9807 at a CTDD of 298 mm. For the outer shell, highest SNR, CC1/2, and R_split_ values were 2.49, 0.7765, and 41.75%, respectively, as obtained from the data processed at a CTDD of 298 mm (Fig 5A), which was close to the actual optimized CTDD.

For HEWL data processed using DirAx, the highest overall SNR, CC1/2, and R_split_ values were 3.10, 0.9026, and 25.98%, respectively (Fig 5B), obtained from the data processed at CTDDs of 297, 292, and 295 mm, respectively, which differed significantly from the actual CTDD. For the outer shell, the highest SNR, CC1/2, and R_split_ values were 1.37, 0.5427, and 75.26%, respectively, as obtained from the data processed at a CTDD of 298 mm (Fig 5B).

For HEWL data processed using XGANDALF, the highest overall and outer-shell SNR, CC1/2, and R_split_ values were 12.66, 0.9944, and 5.98, respectively, both obtained at a CTDD of 298 mm (Fig 5C). For the outer-shell resolution, the highest SNR, CC1/2, and R_split_ values were 5.46, 0.9490, and 18.75, respectively, which were also obtained at a CTDD of 298 mm (Fig 5C). This XGANDALF processing result indicates that the CTDD yielding the highest SNR, CC1/2, and R_split_ values for both the overall and outer shell resolutions were close to the actual CTDD.

CTDD that yields the highest data processing statistics at the overall resolution may vary depending on the indexing algorithm. In contrast, the highest SNR, CC1/2, and R_split_ values at the outer shell resolution were consistently observed at 298 mm across all three indexing algorithms, which is close to the actual CTDD. For all indexing results, the quality of the data processing statistics exhibited a declining trend as the CTDD deviated from the actual CTDD.

**Fig 6:**
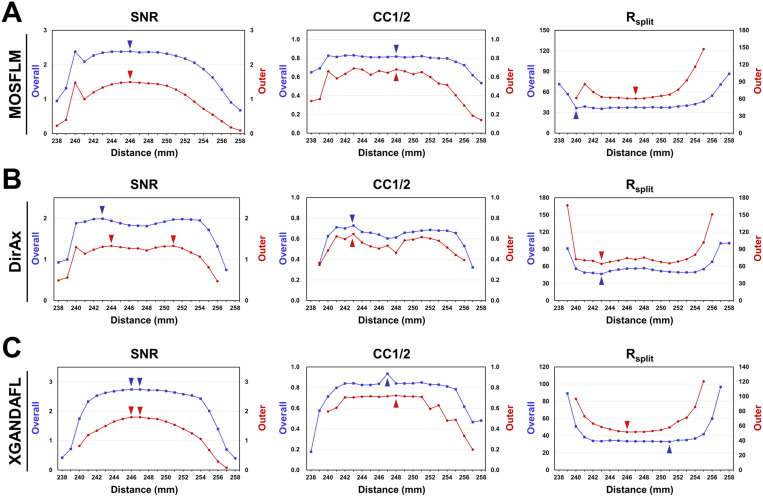
Statistics of GI data processing using different algorithms at various input CTTDs. **(A)** MOSFLM. **(B)** DirAx. **(C)** XGANDALF. Overall (■; blue lines) and outer shell (●; red lines) resolution ranges were 72–1.70 and 1.76–1.70 Å, respectively. Data points with an SNR less than 0 or an Rsplit greater than 200 were excluded from the data plot.

For GI data processed using MOSFLM, the highest overall SNR and R_split_ values were 2.39 and 37.23%, respectively (Fig 6A), which were obtained from the data processed at CTDD of 246 mm and 240 mm, respectively. The highest overall CC1/2 value was 0.8178, obtained with a CTDD of 248 mm. For the outer shell, the highest SNR, CC1/2, and R_split_ values obtained from the data processed at CTDD of 246, 248, and 247 mm were 1.50%, 0.6804%, and 60.71%, respectively. For GI data processed using DirAx, the highest overall SNR, CC1/2, and R_split_ values were 1.99, 0.7274, and 46.71%, respectively (Fig 6B), as obtained from the data processed at a CTDD of 243 mm. For the outer shell, the highest SNR, CC1/2, and R_split_ values were 1.33, 0.6162, and 65.28%, respectively, obtained from the data processed at CTDD of 244/251, 243, and 243 mm, respectively. For GI data processed using XGANDALF, the highest overall SNR, CC1/2, and R_split_ values were 2.74, 0.9342, and 32.85, respectively, obtained at CTDDs of 246/247, 247, and 251 mm, respectively (Fig 6C). For the outer shell, the highest SNR, CC1/2, and R_split_ values were 1.80, 0.7226, and 51.65%, respectively, obtained from the data processed at CTDD of 246/247, 248, and 246 mm, respectively.

Taken together, the highest SNR, CC1/2, and R_split_ values at the outer shell resolution for the HEWL dataset were consistently observed at 298 mm across all three indexing algorithms, which was close to the actual CTDD. As the CTDD deviates further from this range, the quality of these values decreases. For GI data, the highest SNR, CC1/2, and R_split_ values at the overall and outer shell resolutions were not always observed at 248 mm across all three indexing algorithms.

### Statistics of structure determination

**Fig 7:**
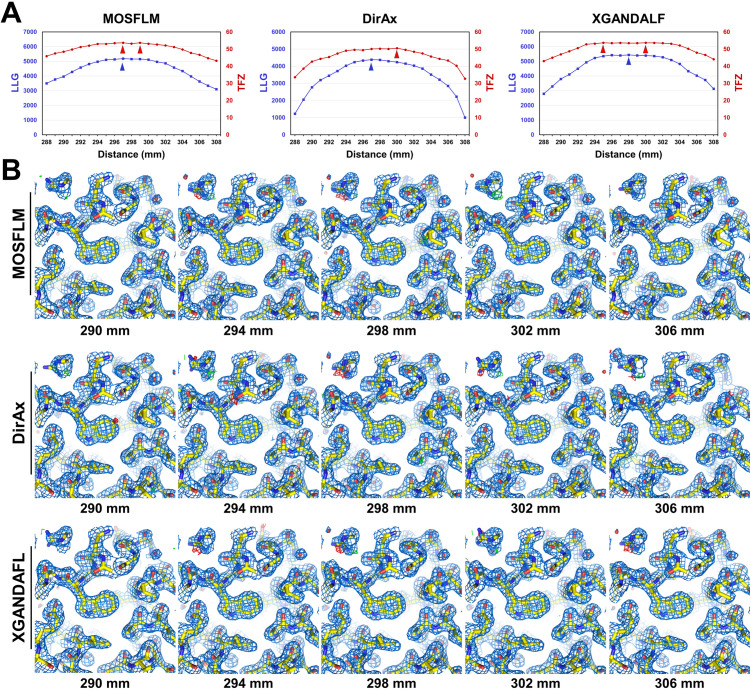
Molecular replacement (MR) solution and electron density maps of HEWL at various input CTDDs. **(A)** Log-likelihood gain (LLG) and translation function Z (TFZ) scores of the MR solution processed by MOSFLM, DirAx, and XGANDALF at various input CTDDs. (B) 2Fo-Fc (marine mesh, 1 σ) and Fo-Fc (green mesh, + 3σ; red mesh, −3σ) electron density maps of HEWL obtained using MOSFLM, DirAx, and XGANDALF at various input CTDDs.

Structure determination statistics were analyzed to investigate the ways in which CTDD processing affected the refinement statistics of the final structures. The phase problems of all HEWL data were solved via MR (Fig 7). For HEWL data processed by MOSFLM, all data exhibited high log-likelihood gain (LLG; > 3102) and final translation function Z (TFZ; > 43) scores, successfully solving the phase problem (Fig 7A). The highest LLG and TFZ values were 5182.750 and 53.7, respectively, which were obtained at CTDDs of 297 and 297/299 mm, respectively. For HEWL data processed by DirAx, all data exhibited high LLG (>1006) and TFZ (>32) values, which also solved the phase problem (Fig 7A). The highest LLG and TFZ values obtained at CTDDs of 297 and 298 mm were 4385.266 and 50.2, respectively. For HEWL data processed by XGANDALF, all data exhibited high LLG (>2791) and TFZ (>43) values, thereby solving the phase problem (Fig 7A). The highest LLG and TFZ values obtained at CTDDs of 298 and 300 mm were 5439.904 and 53.7, respectively.

Electron density maps of HEWL were analyzed at various CTDDs. The quality of all the electron density maps of the entire dataset at CTDDs of 288–308 mm, as determined by MOSFLM, DirAx, and XGANDALF, was clearly observed for model building (Fig 7B). Despite the differences in indexed image numbers and data processing statistics, no significant visual differences were observed in the quality of the electron density maps obtained at various CTDDs. These results indicate that the quality of the electron density maps alone is not a direct criterion for accurately determining the CTDDs.

**Fig 8:**
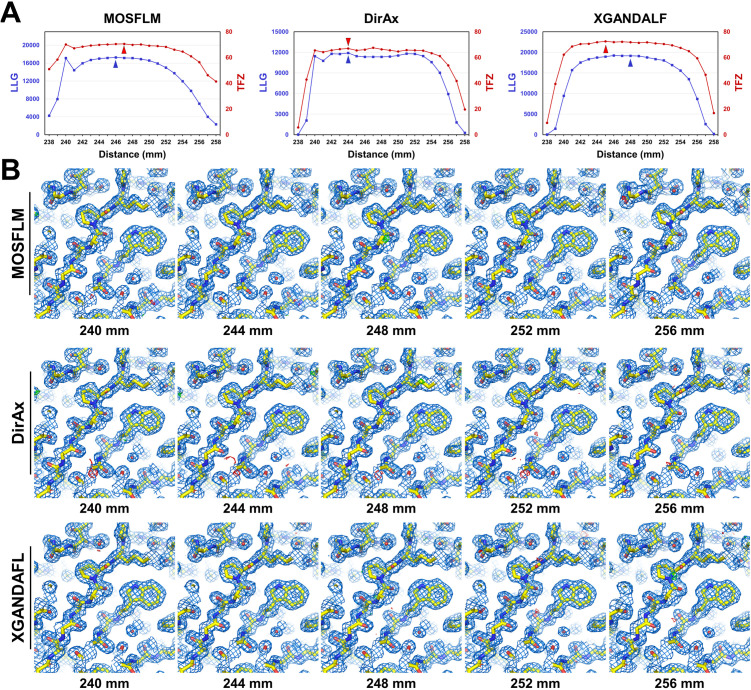
MR solution and electron density maps of GI at various input CTDDs. **(A)** LLG and TFZ scores of the MR solution processed by MOSFLM, DirAx, and XGANDALF at various input CTDDs. (B) 2Fo-Fc (marine mesh, 1 σ) and Fo-Fc (green mesh, + 3σ; red mesh, −3σ) electron density maps of GI obtained using MOSFLM, DirAx, and XGANDALF at various input CTDDs.

The phase problems of all GI data were solved via MR (Fig 8). For the MOSFLM dataset, all GI data exhibited high log-likelihood gain LLG (>2336) values and final TFZ (>41) scores, successfully solving the phase problem (Fig 8A). The highest LLG and TFZ values obtained at CTDDs of 246 and 247 mm were 17268.377 and 70.6, respectively.

For the DirAx dataset, the GI data within the CTDD range of 239–257 mm exhibited high LLG (>1790) and TFZ (>41) values, thus successfully solving the phase problem (Fig 8A). Meanwhile, GI data at CTDDs of 238 and 258 mm exhibited low LLG (<283) and TFZ (<20), respectively. Specifically, data processing at 239 mm did not solve the phasing problem due to the poor data quality. The highest LLG and TFZ values were 11915.300 and 67.0, respectively, which were obtained at a CTDD of 244 mm.

For the XGANDALF dataset, the GI data within the CTDD range of 239–257 mm exhibited high LLG (>1479) and TFZ (>39) values, which solved the phase problem (Fig 8A). Meanwhile, the GI data within the CTDD at 238 and 258 mm exhibited a low LLG (<198 mm) and TFZ (<17 mm), respectively. The highest LLG and TFZ values were 19156.158 and 72.6, respectively, obtained at CTDDs of 248 and 245 mm, respectively.

Electron density maps of GI were analyzed at various CTDDs. The quality of all electron density maps of the entire dataset at CTDDs of 292–304 mm, as determined by MOSFLM, DirAx, and XGANDALF, was clearly observed for model building (Fig 8B). Despite the differences in the indexed image numbers and data processing statistics, no significant visual differences were observed in the quality of the electron density maps obtained at various CTDDs. These results also indicated that the electron density maps could not provide direct information for the corrected CTDDs.

### Statistics of structure refinement

**Fig 9:**
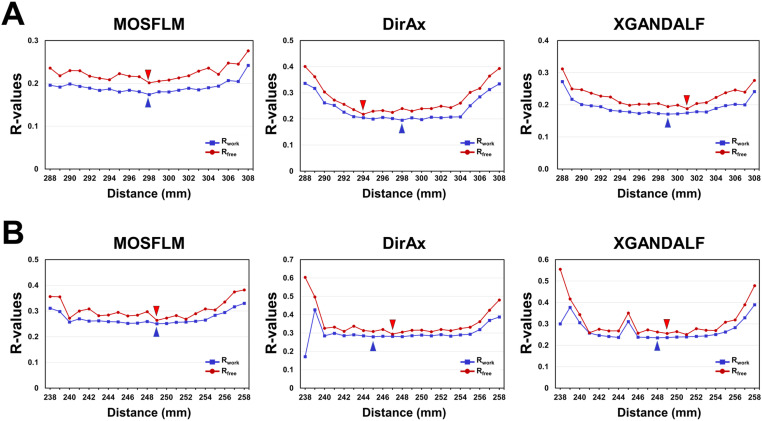
Structure determination statistics of HEWL and GI. Structure determination statistics of **(A)** HEWL and **(B)** GI processed by MOSFLM, DirAx, and XGANDALF at various input CTDDs. R_free_/R_work_ values of HEWL and GI in the resolution ranges of 39.52–1.80 and 47.12–1.70 Å, respectivley, are shown. The lowest R_work_ and R_free_ values are indicated by blue and red triangles, respectively.

Structural refinement of the processed HEWL dataset was performed at a resolution of 1.8 Å. For the MOSFLM dataset, structural refinement showed that the R_work_ and R_free_ values of HEWL at a CTDD of 298 mm were 17.38 and 20.15%, respectively, which were lower than those for the other datasets (Fig 9A). All HEWL structures within the CTDD range of 288–308 mm exhibited R_work_ and R_free_ values less than 25 and 28%, respectively, falling within the acceptable range for general protein crystallography. For the DirAx dataset, structural refinement showed the lowest R_work_ value of 19.52% at a CTDD of 298 mm (Fig 9A). The lowest R_free_ value of HEWL was 21.80% at a CTDD of 294 mm. HEWL structures within the CTDD range of 291–304 mm exhibited R_work_ and R_free_ values less than 25 and 30%, respectively. For the XGANDALF dataset, HEWL structural refinement showed the lowest R_work_ value of 17.09% at a CTDD of 299 mm (Fig 9A). The lowest R_free_ value of HEWL was 18.88% at a CTDD of 301 mm. HEWL structures within the CTDD range of 289–308 mm exhibited R_work_ and R_free_ values less than 25 and 28%, respectively.

Structural refinement of the processed GI dataset was performed at a resolution of 1.7 Å. For the MOSFLM dataset, GI structural refinement revealed that the R_work_ and R_free_ values of HEWL at a CTDD of 249 mm were 25.03 and 26.41% lower, respectively, than those for the other datasets (Fig 9B). All GI structures within the CTDD range of 240–253 mm exhibited R_free_ values less than 30%, falling within the accepted range for general protein crystallography. For the DirAx dataset, GI structural refinement resulted in the lowest R_work_ value of 28.04% at a CTDD of 248 mm (Fig 9B). The lowest R_free_ value of HEWL was 29.47% at a CTDD of 247 mm (Fig 9B). For the XGANDALF dataset, GI structural refinement showed the lowest R_work_ value of 23.51% at a CTDD of 248 mm. The lowest R_free_ of HEWL was 25.52% at a CTDD of 249 mm (Fig 9B). Overall, acceptable R_work_ and R_free_ values of GI were obtained at near-optimized CTDDs. R_work_ and R_free_ values of GI data were also relatively high due to the insufficient indexed data volume [51].

## Discussion

Structure determination via SX provides biologically relevant room-temperature structures better than other structural biology techniques. In SX, CTDD variations can occur during the delivery of various crystal samples to the X-ray interaction point using a sample delivery system. To evaluate the impact of CTDD accuracy on data quality, data processing and structure determination of HEWL and GI were performed at various CTDDs using three different indexing algorithms.

The CTDD that yielded the maximum number of indexed images of both HEWL and GI using MOSFLM, DirAx, and XGANDALF differed from the actual CTDD. Additionally, the CTDD that resulted in the highest number of indexed diffraction images varied based on the indexing algorithm used. Therefore, CTDD at which the maximum number of images are indexed may not serve as a reference for the optimal CTDD. Indexing tendencies, indexable CTDD range, and number of indexed diffraction patterns varied depending on the indexing algorithm used. These results suggest that the indexing outcomes also vary depending on the indexing algorithm used during diffraction data processing.

Analysis of unit cell distribution using all diffraction data processed by MOSFLM, DirAx, and XGANDALF revealed a Gaussian distribution in the CTDD range of 298–300 mm for HEWL and 248–250 mm for GI, which were close to the actual optimized CTDD range. When the CTDD was lower or higher than that in the experimental setup, the unit cell dimensions shortened or lengthened, respectively, and Gaussian distribution of the unit cell deteriorated. Therefore, this study investigated the unit cell distribution of indexed images to track the actual CTDD, similar to previous reports [21,52].

Although the data processing results varied depending on the indexing algorithm used, the highest data quality indicators, such as SNR, R_split_, and CC values for outer shell resolution, were observed near the CTDD range of 298–299 mm for HEWL and 248–249 mm for GI, which were further used to estimate the actual CTDD. In the data statistics plots for SNR, R_split_, R_free_/R_work_, and B-factor, some values did not exhibit a uniform overall trend, indicating a static error. Therefore, in these statistical plots, overall trend was considered with respect to distance rather than the absolute value of each data point.

During structure determination, all indexing algorithms successfully provided MR solutions for data processing in the CTDD range of 288–308 mm for HEWL and 238–258 mm for GI, except for the dataset processed by DirAx at a CTDD of 288 mm. This finding indicates that the CTDD deviations by a few millimeters did not pose any issues in solving the phase problem. Therefore, obtaining an MR solution using processed SX data from actual experiments does not imply that the CTDDs are accurate.

After structural refinement, data quality varied depending on the indexing algorithm used, and optimal CTDDs for producing the best statistical values differed among the algorithms. For example, lowest R_free_ values were obtained at a CTDD of 298 mm with MOSFLM, whereas the best overall statistics were obtained at a CTDD of 301 mm with XGANDALF. These findings suggest that the optimal structure refinement results not only vary according to the indexing algorithm but also differ by a few millimeters from the actual experimental distance. These differences were possibly due to CTDD variations during sample delivery and the indexing algorithm type. In terms of optimal structure refinement statistics, although the best CTDD was between 298 and 300 mm, all datasets processed by MOSFLM, DirAx, and XGANDALF in the CTDD ranges of 288–308, 292–304, and 289–307 mm, respectively, showed the generally accepted crystallographic R-values (R_work_ < 25% and R_free_ < 30%). This finding suggests that structure determination is possible with an inaccurate CTDD.

In an ideal experiment, correct CTDD is predetermined and applied directly to data processing, thereby decreasing the need for the careful consideration of CTDD. However, CTDD varies in SX as the sample position fluctuates during sample delivery. The experimental results of this study provide preliminary insights into CTDD tolerance. If the sample position shifts during SX data collection, optimal CTDDs can be determined based on the unit cell distribution, data processing, and refinement results of the data at various intervals.

The results of this study also suggest that the processed data varies based on the indexing algorithm and input parameters during data processing, thereby affecting the statistics of data processing and structure determination. Therefore, rather than the absolute statistical values, trends in data processing results related to CTDD are more important for data interpretation. Additionally, data processing trends vary depending on the experimental setup and crystal characteristics, such as the unit cell distribution, quality, and resolution. Therefore, future studies should investigate the CTDD tolerance using different experimental setups and crystal types. In this study, only the datasets obtained using the syringe and viscous medium-based sample delivery method were used. Future SX studies should evaluate the impacts of different sample delivery methods on CTDDs to further enhance data processing.

## Conclusion

In this study, HEWL and GI diffraction data were processed using the CrystFEL program at various CTDDs with three different indexing algorithms to understand the impact of CTDD accuracy on SX data processing. Although small differences in CTDDs (on the millimeter scale) did not affect MR phasing and structural determination, inaccurate CTDDs resulted in relatively high R values. Therefore, application of correct CTDDs is essential for accurate structure determination. Accurate CTDD tracking can be achieved by considering the unit cell distribution, overall and highest shell SNR, R_split_, CC, and structure refinement statistics. Overall, CTDD analysis results of this study provide crucial insights for effective SX data processing.

## Supporting information

S1 Fig Unit cell distribution of the indexed HEWL images processed by MOSFLM at various input CTDDs. (DOCX)

S2 Fig Unit cell distribution of the indexed HEWL images processed by DirAx at various input CTDDs. (DOCX)

S3 Fig Unit cell distribution of the indexed HEWL images processed by XGANDALF at various input CTDDs. (DOCX)

S4 Fig Unit cell distribution of the indexed GI images processed by MOSFLM at various input CTDDs. (DOCX)

S5 Fig Unit cell distribution of the indexed GI images processed by DirAx at various input CTDDs. (DOCX)

S6 Fig Unit cell distribution of the indexed GI images processed by XGANDALF at various input CTDDs. (DOCX)
