# Supplementary material for: Effect of crystal-to-detector distance shift on data processing in serial crystallography
Source: PLoS One. 2025 Jun 26;20(6):e0327019. doi: 10.1371/journal.pone.0327019 (PMC12200686; doi:10.1371/journal.pone.0327019)
Supplement: S4 Fig — (DOCX) [file pone.0327019.s004.docx]

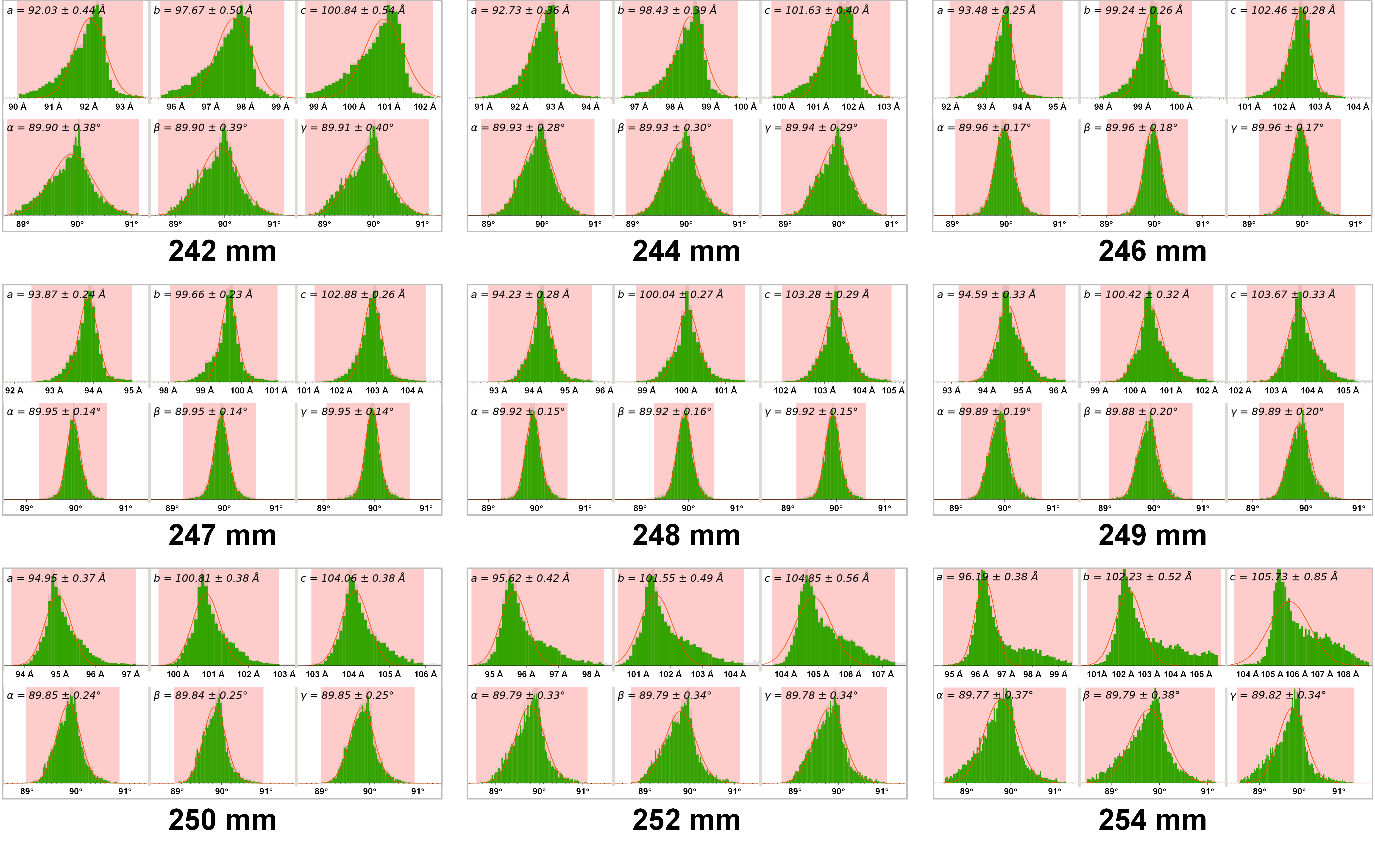


**S4 Fig**. Unit cell distribution of the indexed GI images processed by MOSFLM at various input CTDDs.
