# Supplementary figures and images for: Effect of crystal-to-detector distance shift on data processing in serial crystallography
Source: PLoS One. 2025 Jun 26;20(6):e0327019. doi: 10.1371/journal.pone.0327019 (PMC12200686; doi:10.1371/journal.pone.0327019)

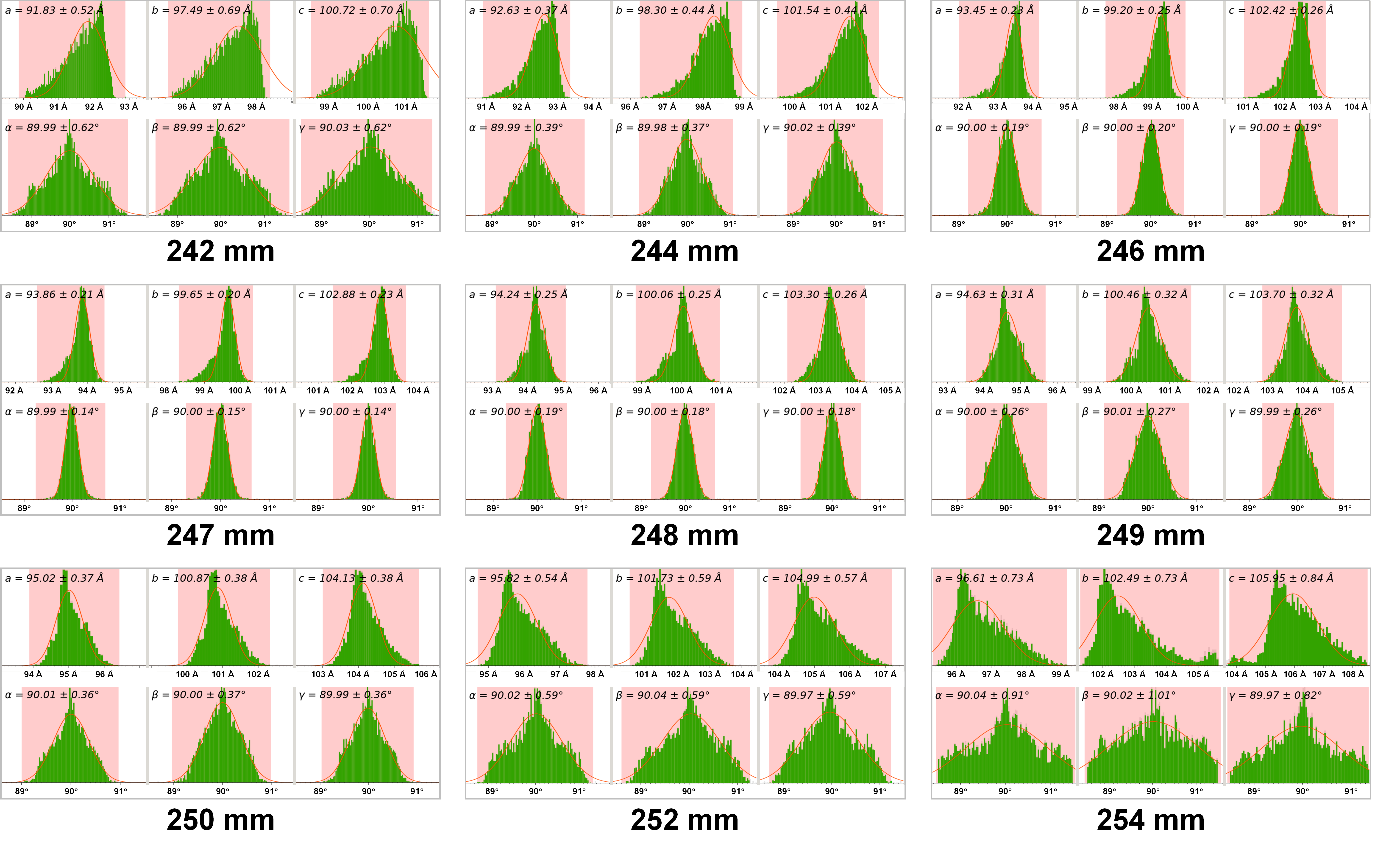


**S5 Fig**. Unit cell distribution of the indexed GI images processed by DirAx at various input CTDDs.

Supplement: S5 Fig — (DOCX) [file pone.0327019.s005.docx]
